# Experimental Studies on Adaptive-Passive Symmetrical Granular Damper Operation

**DOI:** 10.3390/ma15176170

**Published:** 2022-09-05

**Authors:** Mateusz Żurawski, Robert Zalewski

**Affiliations:** Institute of Machine Design Fundamentals, Warsaw University of Technology, 02-524 Warsaw, Poland

**Keywords:** granular structures, vacuum-packed particles, granular damper, experimental research, semi-active damper

## Abstract

This paper presents experimental studies on a controllable granular damper, whose dissipative properties are provided by the friction phenomenon occuring between loose granular material. In addition, in order to adjust to the current trends in vibration suppression, we built a semi-active device, controlled by a single parameter—underpressure. Such granular structures subjected to underpressure are called Vacuum-Packed Particles. The first section presents the state of the art. A brief description of the most often used intelligent and smart materials for the manufacture of dampers is presented. The main advantages of the proposed device are a simple structure, low construction cost, symmetrical principle of operation, and the ability to change the characteristics of the damper by quickly and suddenly changing the negative pressure inside the granular core. The second section provides a detailed description of the construction and operation principles of the original symmetrical granular damper. A description of its application in the laboratory research test stand is also provided. The third section presents the results of the experimental studies including the recorded damping characteristics of the investigated damper. The effectiveness of the ethylene–propylene–diene grains’ application is presented. The two parameters of underpressure and frequency of excitation were considered during the empirical tests. The influence of the system parameters on its global dissipative behavior is discussed in detail. The damper operation characteristics are close to linear, which is positive information from the point of view of the potential adaptive-passive control process. Brief conclusions and the prospective application of vacuum-packed particle dampers are presented in the final section.

## 1. Introduction

Observing the engineering environment, it can be concluded that vibrations appear almost everywhere. For reasons of safety or comfort, attention should be paid to mechanical vibrations directly affecting devices or structures. The development of materials and manufacturing technology allows for the construction of ever lighter structures [1]. Their low mass very often makes them more susceptible to external factors. That is why the role of vibration eliminators in modern systems is so important [2,3,4]. New design requirements concerning, inter alia, safety, environmental protection, and economic aspects force changes both in the design of vibration dampers and in the development of new methods of vibration suppression. Currently, many examples can be distinguished in which the phenomenon of resonance is not the only dangerous one. There are several forms of excitation. For example, each vehicle is exposed to varying weather conditions and a variety of road surfaces [5]. It is obvious that the classical damper cannot be used in both cases. The simplest passive damper [6] has permanent dissipation properties, so it is not possible to adjust the damping force to the current external conditions. For such a case, smart and intelligent materials should be taken into account. Applying the previously mentioned group of structures makes it possible to change the damping properties of vibration reduction devices. The advantages of this type of solution are so significant that more often active or semi-active attenuators can be found in practical engineering applications. At the same time complex laboratory tests are constantly being carried out to improve this type of devices.

According to the definitions presented in [7], passive vibration dampers are relatively simple technical devices. They are used to change the stiffness and damping of the structure in the desired way and, at the same time, do not require an external power supply. Characteristic features of this type of absorber include permanent dissipation properties. Due to the previously mentioned simplicity of construction, they are still the most often used vibration reduction systems [8]. Another example is the use of the double-layer permanent-magnet buffer [9]. The authors showed the possibility of damping the vibrations caused by the impact load of the launcher.

Based on [10], active vibration damping consists of the dissipation of vibration energy through the application of appropriate external forms of energy to the investigated dynamical system. Generally, active dampers allow for a dynamic change in the damping characteristics depending on the various conditions. The entire process is managed by a controller which, based on sensor data, sets the appropriate actuator damping values. The active vibration damping system [10] has the greatest possibilities, but at the same time requires the use of additional equipment and the supply of energy from external sources. In the case of such types of system, intelligent materials are often used.

Semi-active vibration damping [7] does not introduce an additional energy into the system. It enables changing the dissipation properties of the actuator responsible for efficient damping. In fact, the system can adapt to the changing exciting load.

Scientists and researchers aim to propose novel designs of devices that could be an alternative to the commonly used controlling absorbers. Several papers [11,12,13,14] present an approach in which mechanical vibrations were damped by means of an innovative adaptive impact damper. The main factor reducing vibrations is the possibility of mass movement inside a container of variable volume. This type of damper is effective especially for resonant frequencies or free vibrations. Another adaptive device was described in [15,16]. The main innovation of that approach is the ability to control the size of the valve in the cylinder which directly affects the effectiveness of shock absorption. A further example of devices that can change their damping properties as a result of external factors are Vacuum-Packed Particles. Detailed descriptions of this type of absorber can be found in [17,18,19,20,21]. The discussed damping attenuators are characterized by a granular core with variable pressure inside. By changing the pressure, the contact forces between the grains can be controlled [22,23,24,25]. As a result, the characteristics of the hysteresis function, describing the dependence of the forces generated by the devices as the effect of compression and tension are changed. The main disadvantage of the described devices is an effective damping only in the compression stage of the core. Therefore, the authors of this paper propose an approach that allows obtaining the symmetrical characteristics of the force-displacement hysteresis loops. Such a solution allows for the extension of the applicability of this type of device.

To further emphasize the importance of symmetry in mechanical engineering, several works based on solutions that increase the reliability of the structure by ensuring symmetry should be cited [26,27,28,29,30]. A frequent effect of the influence of excitation on a structure is its unforeseen destruction. Therefore, reliability analyses of structures characterized by a symmetrical structure are carried out. These are implemented through the innovative method for symmetry representations and elastic redundancy for members of tensegrity structures [26]. An analogous approach to modeling can be found in systems called origami structures. New types of thin-walled origami tube based on the Kresling origami pattern have been proposed [27]. These studies were extended and improved through the use of Convolutional Neural Networks [28]. Engineering applications where there are vibration and bifurcation problems and the stability of systems exhibiting symmetry have been studied using group theory [29] and layered space grids [30]. An interesting issue is the effect of nanomaterials and research fibers on the mechanical properties of symmetrical polymer pomposites [31]. In this approach, the impact and tensile properties of the polypropylene nanocomposites with graphene nanosheets, nanoclay, and basalt fibers were explored.

The paper outline is as follows. After the introduction to the subject, the construction of a symmetrical granular damper is presented and described in detail. Then, the test stand and experimental results are presented. The experimental analysis was performed for several different parameters of the underpressure and the excitation frequency. Finally, the results are compared, and the general characteristics of the presented novel device are determined. The paper is summarized with synthetic conclusions.

## 2. Prototype of the Symmetrical Granular Damper

**Damper Construction**

For many decades, engineers and scientists have been trying to propose newer solutions, whose principles are often very complicated. Therefore, the mechanical systems and the materials have become very advanced in their structure. This offers great opportunities to design adaptive, active, or semi-active devices. As a result, the discussed solutions are very expensive or have a very narrow range of applicability. One of the main assumptions of this paper was to propose an innovative device that would be characterized by a simple structure with low cost, features of adaptability, and a wide range of application possibilities.

The following description presents a vibration damper with a granular core having variable and controllable dissipation properties. The system confists of a rigid cylinder encased by a sealed granular core made of Vacuum-Packed Particles (VPP). The ability to control the pressure in the granular container allows changing the macroscopic damping properties of the device. This solution enables obtaining a symmetrical response characteristic and changable dissipation properties depending on the pressure inside the core and the initial tension of the springs. The design and principle of operation of the presented innovative symmetrical granular damper have been patented [32].

**Figure 1:**
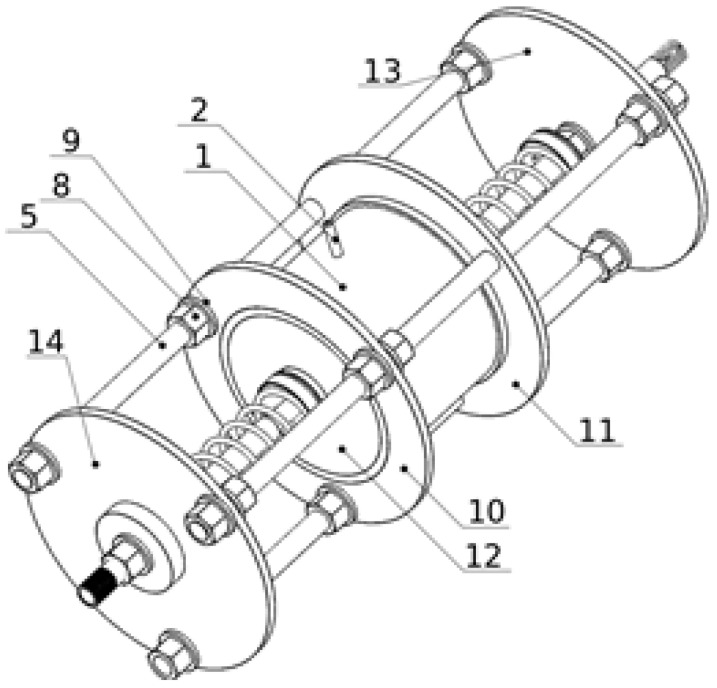
Scheme 1 of the symmetrical granular damper.

**Figure 2:**
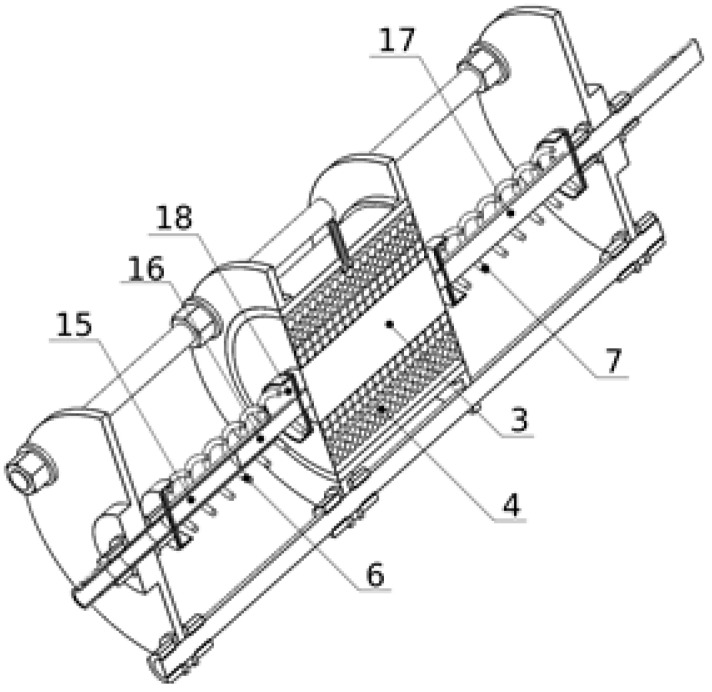
Scheme 2 of the symmetrical granular damper.

Depicted in Figure 1 and Figure 2, the vibration damper with variable dissipation properties consists of a plastic cylinder (1) with flanges (10, 11) attached at its outer periphery to its opposite edges. The flanges (10, 11) of the cylinder (1) are rigidly connected by three parallel bars (5) to the first and the second retaining discs (13, 14) located at the ends of the damper. In the sealed chamber of the cylinder (1), there is a ring-shaped granular core (4) made of VPP. Inside the granular core (4) there is a rigid cylindrical ring (3). The valve (2) is mounted in the casing of the cylinder (1) to regulate the partial vacuum inside the granular core (4). The sealing of the cylinder chamber (1) consists of an elastic annular carcass (12) made of rubber, fixed on the opposite sides of the cylinder (1), between its edges, and the ends of the cylindrical core (3). The cylindrical core (3) is coaxially connected to a bar (17) slidably mounted on the first retaining disc (13). The granular core (4) transmits the linear load exerted on the cylindrical core (3). In the axis of the cylinder (1), on the opposite sides of the cylindrical core (3), there are two springs (6, 7) provided to compensate the semi-plastic deformation of the granular core (4) in the axial direction under the influence of the linear load. The spring element (6) is mounted between the cylindrical core (3) and the first retaining disk (13). The second spring (7) is located between the cylindrical core (3) and the second retaining disk (14). The ends of the springs (6, 7) are supported in the spring seats (18) setting their axial position. The rod (17) is positioned in the axis of the first spring (6). In the axis of the second spring (7), there is a telescopic guide element, the first member of which (16) is attached to the cylindrical body (3), and the second member (15) is attached to the second guide disc (14). Nuts (8) with washers (9) were used for the bolted connections of the cylinder (1) and the retaining discs (13, 14) with the rods (5).

**Figure 3:**
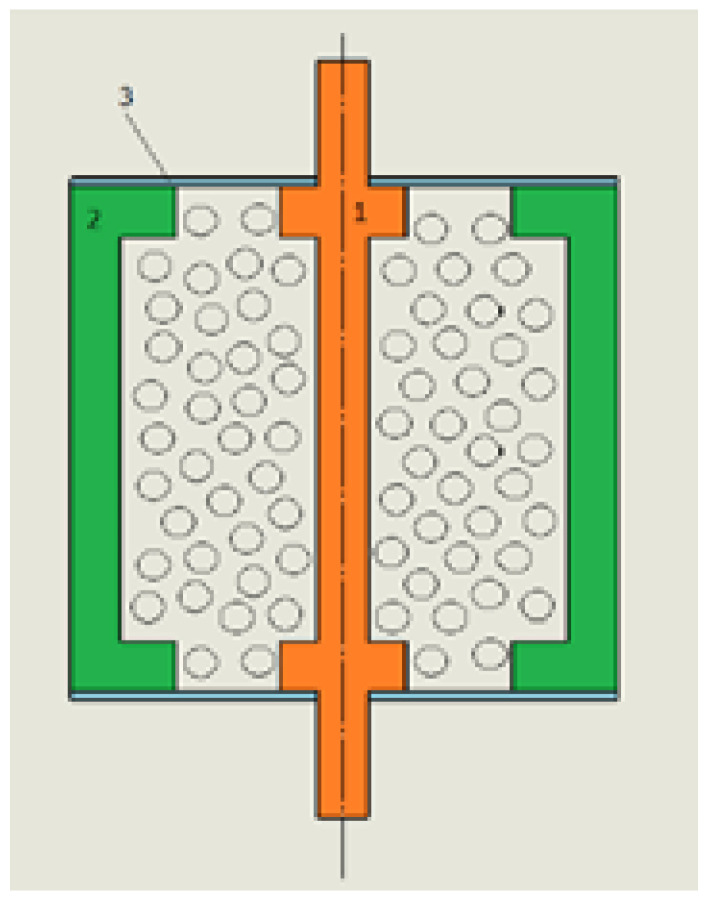
Scheme of the principle of the operation—initial stage.

**Figure 4:**
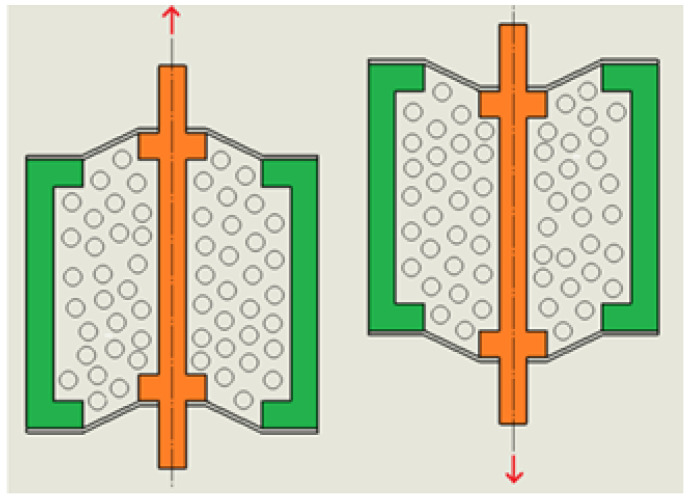
Scheme of the principle of the operation—operating stage.

For a detailed description of the operating principle of the proposed device, a simplified diagram is presented in Figure 3 and Figure 4. The granular material is located between a fixed outer sleeve (2) and a movable inner sleeve (1). The thin silicone sealing plates (3) are glued to the upper and lower parts of the device. Such a solution protects the loose material from falling out, and due to its elastic properties, it allows maintaining full tightness during the movement of the central sleeve.

The stationary outer sleeve (2) and the movable inner sleeve (1) simulate the phenomenon of shearing.

Existing devices [13,14,17,18,21] reflect the effective ability of the mechanical damping. Despite many advantages, the functionality of the aforementioned structures is subject to significant limitations. This causes the limited use of classic granular dampers in technical applications. The main disadvantage of the existing solutions is that they only work effectively in one direction when the granular core is compressed.The proposed design eliminates the disadvantages of classic approaches, thus extending the scope of applicability in mechanical structures subjected to various excitations.

**Figure 5:**
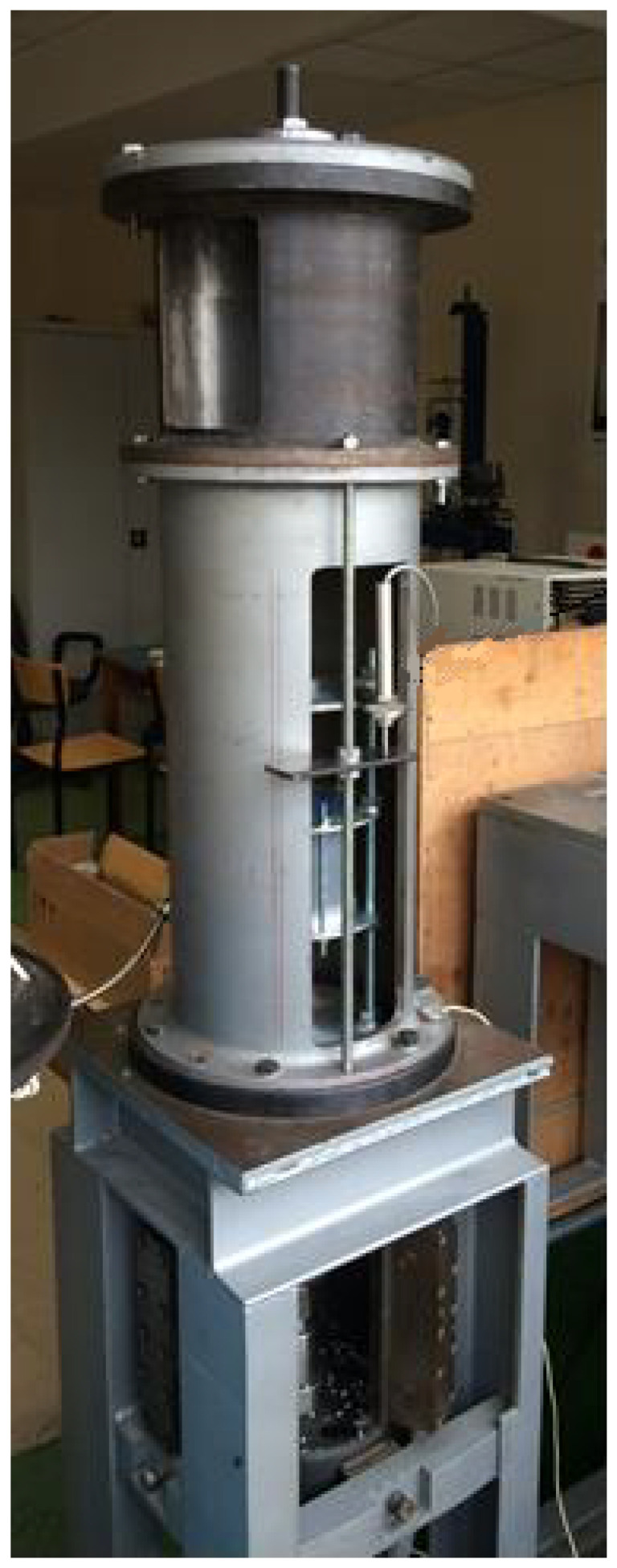
Test stand.

The relatively simple design assumptions of the damper made it possible to manufacture all the elements and test the device on a testing machine in order to confirm the previously mentioned assumptions about the symmetrical damping characteristics. The test stand (Figure 5) consists of an electric motor connected to the disk, to which an eccentrical pusher was attached. Hence, the reciprocating movement of the device was achieved.

**Figure 6:**
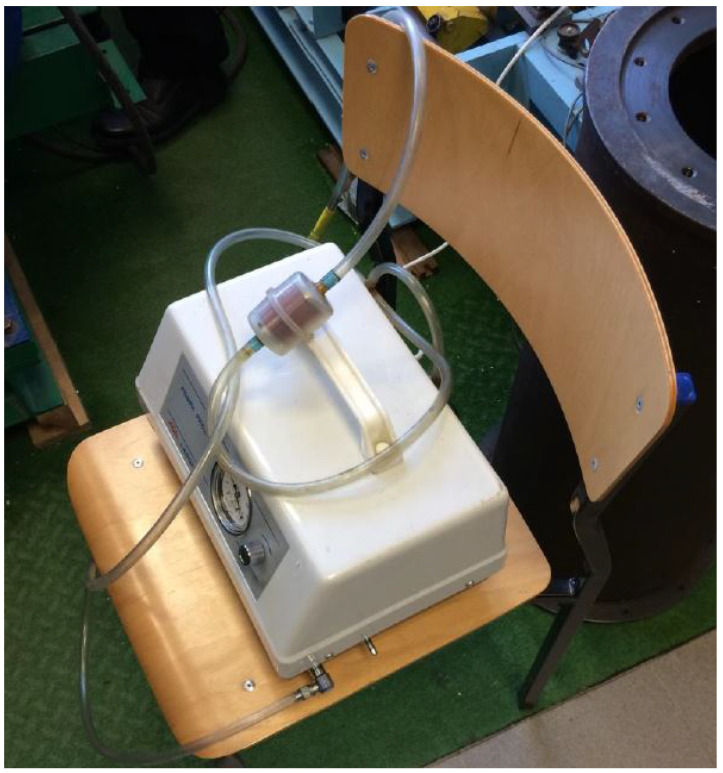
AGA Labor vacuum pump.

With the applied movement mechanism a sine kinematic excitation rule was considered in which both amplitude and frequency could be adjusted. In the conducted research, two quantities were recorded: force and displacement. The force was measured by a piezoelectric sensor installed in the lower part of the test stand, while the displacement measurement was performed by an inductive displacement sensor. In the case of the tests carried out on a granular damper, the two parameters were regulated. The amplitude was constant at 5 mm. The frequency of the motor’s rotation (*f*) was set by means of an inverter. On the other hand, the underpressure was generated with the use of a vacuum pump made by AGA Labor (Figure 6).

## 3. Experimental Results

In order to ensure the comprehensiveness of the tests, experiments were carried out for different rotational speeds and for variable values of partial vacuum inside the granular core. For the selected types of grains, measurements were carried out for five rotational frequencies (Table 1).

The excitation amplitude was constant A=5 [mm]. For such assumptions, the function describing the harmonic input takes the form:

(1) u=Asin(2πft)

During the laboratory tests of the Symmetrical Vacuum-Packed Particle Damper (SVPPD) the most important aspect was investigating the influence of the partial vacuum value, generated inside the granular system—a factor that directly influences the dissipation properties. The internal partial vacuum was changed from 0 to 0.09 [MPa] by means of a vacuum pump (Table 2). In order to investigate the influence of the pressure on the damping characteristics as precisely as possible, the pressure was changed by 0.01 [MPa].

**Figure 7:**
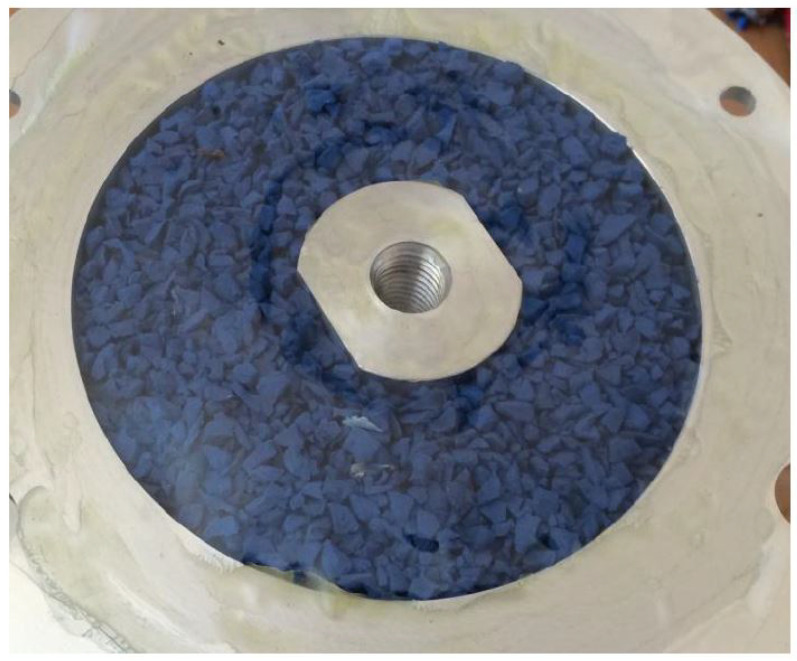
SVPP damper with EPDM grains.

The SVPPD was filled with ethylene–propylene–diene grains (EPDM). The low price and the ability to choose the size and color has contributed to the wide use of EPDM granules (Figure 7) in the construction of various types of facilities as a top layer or filling for football fields, running tracks, and playgrounds for children.

The physical and mechanical properties of the applied grains are shown in Table 3.

**Figure 8:**
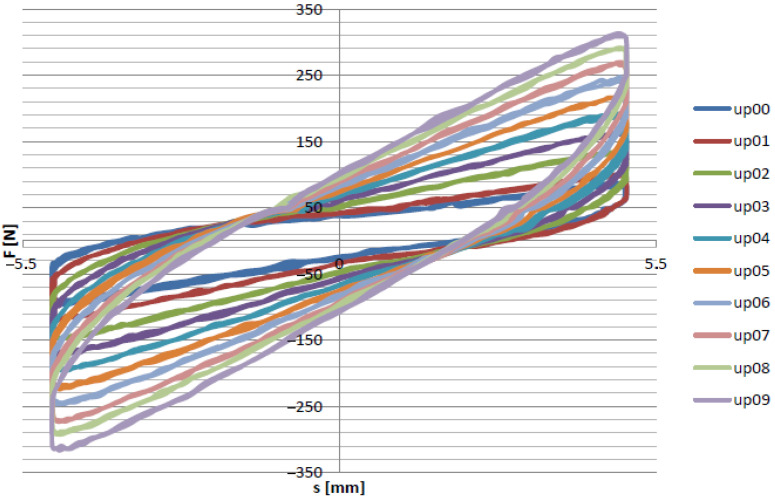
Force/displacement curve for frequency $f_{1}$.

From the experimental tests, it was possible to analyze both the influence of the rotational speed on the damping properties of the device and the influence of underpressure on the increase in the damping force. The main goal of the first group of experiments was to determine the characteristics of the damping force as a function of displacement for the selected frequency $f_{1}$. The results are shown in Figure 8.

**Figure 9:**
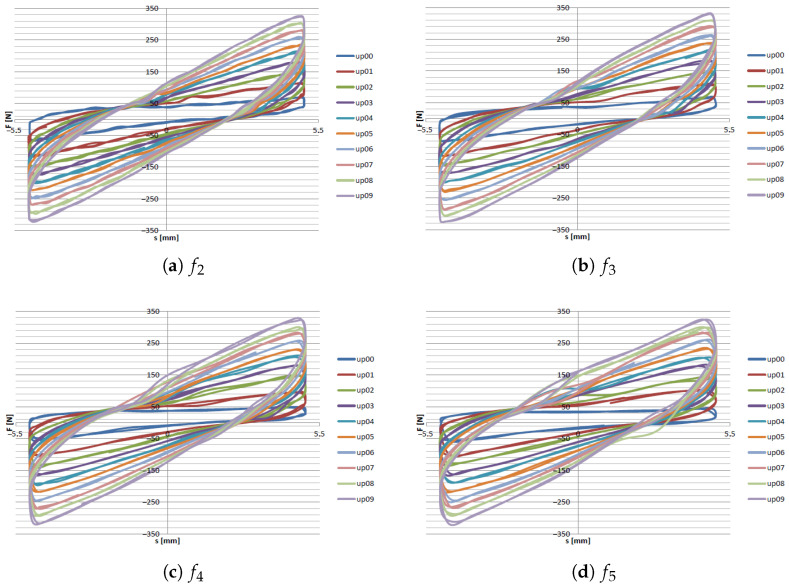
Force/displacement curves for various frequencies $f_{2}$→$f_{5}$.

Figure 8 presents the recorded force-displacement hysteresis loops for the selected loading frequency and underpressure values. The shapes of the recorded data were symmetrical. The maximal value achieved by the SVPPD force was approximatelly 325 N, which was observed for the highest value of the internal underpressure. Decreasing the partial vacuum caused a decrease in the maximum value of the force. The results of the experimental tests for the remaining values of the excitation frequency are presented in Figure 9.

Regardless of the rotational speed, the crucial increase in damping force values was visible during the increase in the applied underpressure. It is also very important that the character was symmetrical during the shear of the granular media. By carrying out tests for various excitation frequencies, it was also possible to investigate the influence of this parameter on the values of the damping force. The figures below show the influence of the rotational speed on the recorded forces for the SVPPD.

**Figure 10:**
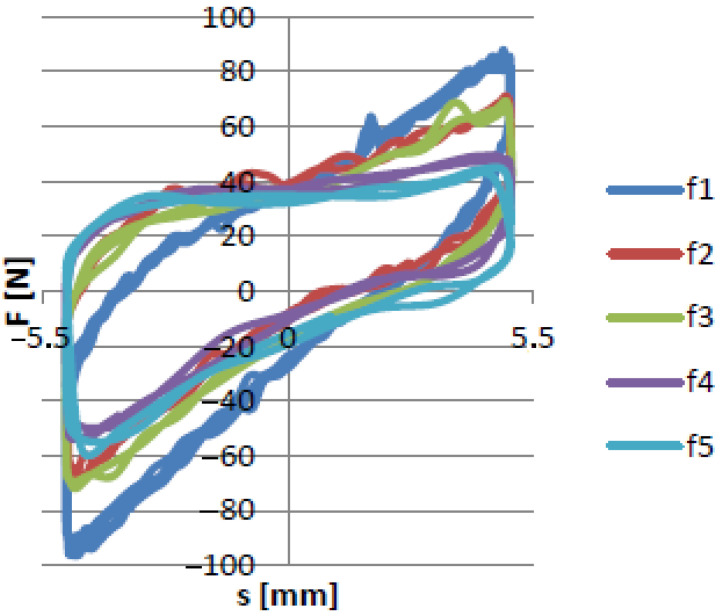
Force/displacement characteristics for various rotational speeds for up00 underpressure.

When analyzing the above figure, it should be noted that in the case of the zero underpressure value (Figure 10), some differences were noticeable in the values and the characteristics. This type of phenomenon can be caused by the shear of the granulate (as opposed to the compression/stretching) and the gravitational forces that result in the irregular distribution of the grains. The characteristics began to coincide with the higher stiffness of the structure (higher underpressure).

**Figure 11:**
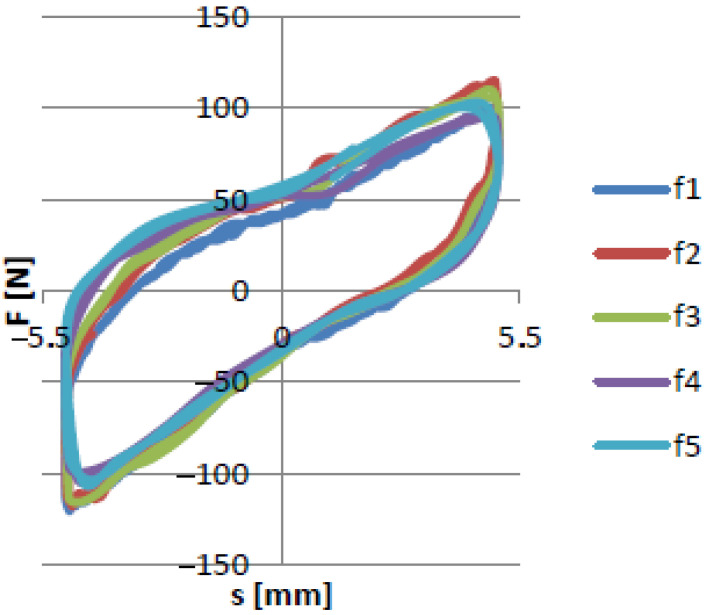
Force/displacement characteristics for various rotational speeds for up01 underpressure.

**Figure 12:**
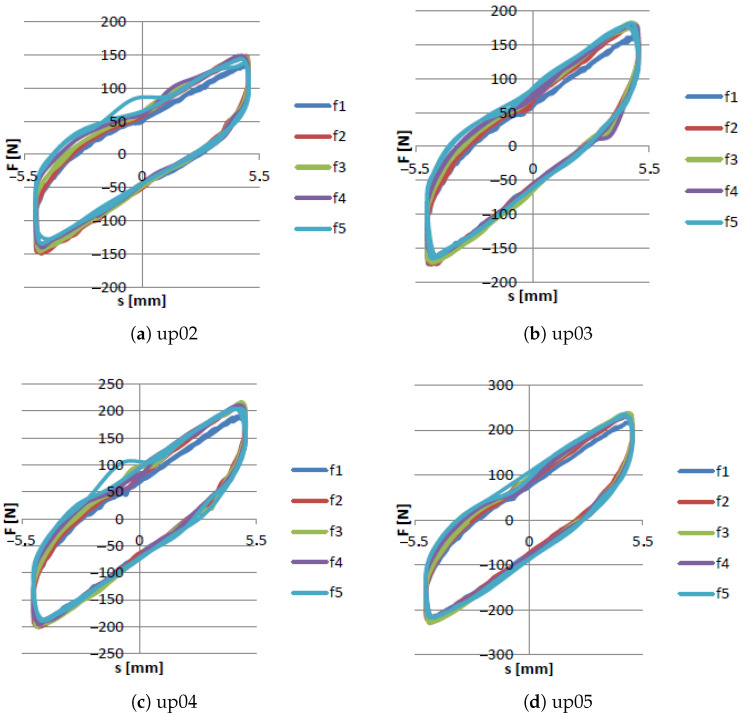

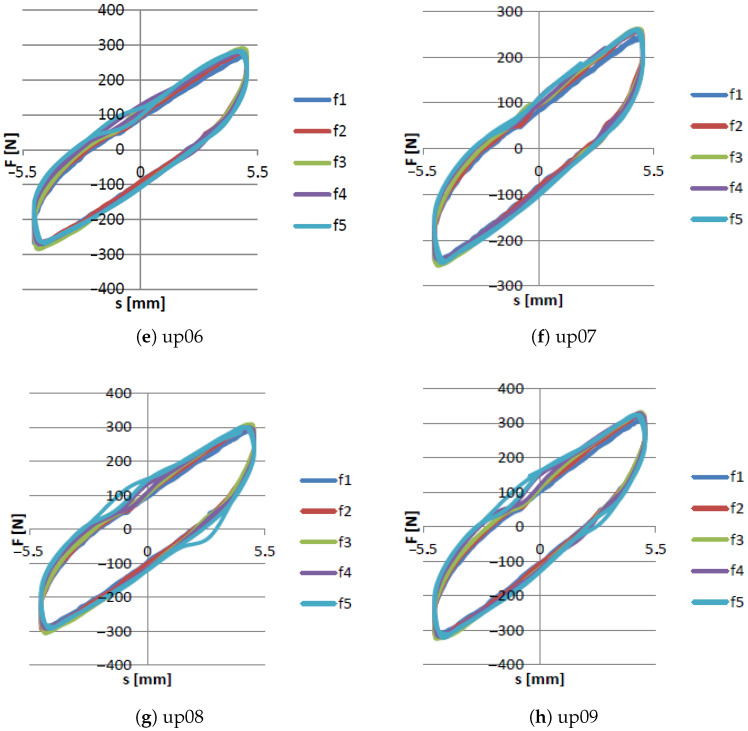
Force/displacement characteristics for various rotational speeds for up02 → up09 underpressure.

**Figure 13:**
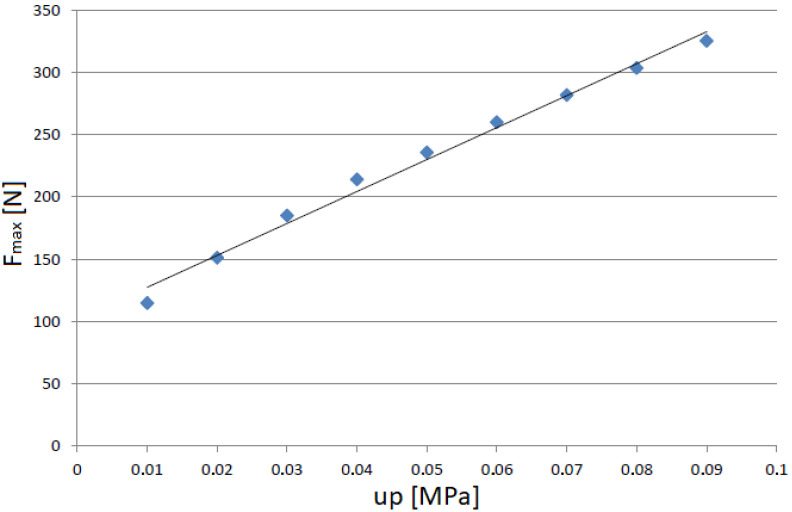
Force/underpressure characteristics for $f_{2}$ rotational speed.

Figure 11 and Figure 12 show that the excitation frequency in the case of the EPDM material had no major influence on the dissipation properties of the damper when the higher values of partial vacuum were generated inside the damper. Based on such an observation it can be concluded that the EPDM granules allow obtaining high damping forces in each case. Moreover, it can be observed that the shapes of the recorded hysteresis loops were similar. The dependence between underpressure and maximal dissipation force is presented in Figure 13.

The obtained results were a function close to linear and had values between 115 [N] and 324 [N]. Analogous dependencies for the remaining excitation frequency cases ($f_{3}$, $f_{4}$, and $f_{5}$) were also characterized by similar linearities. This means that the presented structure can be used in a wide range of work. In the case of semi-active dampers, this is a strong advantage, especially in practical engineering applications.

## 4. Summary

The characteristics of vibrations for an innovative symmetrical Vacuum-Packed Particles absorber were experimentally investigated. The process of pumping out the air from the sealed granular core allowed changing the internal grain structure causing jamming of the loose material being placed inside the damper. This is how the range of deformations of the granular core can be controlled. This phenomenon enabled control of the symmetric dissipation forces. Experimental tests were carried out on the physical model of a granular shear damper. The results presented force-displacement hysteresis loops for excitation frequency and underpressure values. Regardless of the rotational speed, the crucial increase in damping forces values was visible for various implemented underpressures. It should be noticed, that for the zero underpressure state, the various frequencies had a nonlinear influence on the damping forces. However, generally, the change in the underpressure in the granular core caused an almost linear change in the forces generated by the symmetrical damper. The novelty of the presented original damper prototype was based on the use of the shear phenomenon of loose materials and thus obtaining a symmetrical dissipation characteristic, different from the case of the compression and tension of granular structures. Since the shear effect between the grains is the same regardless of the direction of the damper movement, the dissipation characteristic should remain symmetrical. Experimental tests carried out on an experimental stand confirmed the theoretical assumptions.

The novel symmetrical granular damper can be treated as an alternative to current semi-active devices and can be implemented at low-cost in an engineering environment. Many mechanical systems are subject to variable excitation. This cause a multiple analysis of the optimal parameters that would allow for the most effective damping of mechanical vibrations. The proposed damper design may constitute the basis for a device included in cyberphysical systems. These are systems based on the measurement of significant dynamic quantities and their use in advanced numerical analysis, often carried out in the data cloud and enriched with artificial intelligence algorithms; then, with appropriate mechatronic controllers, the process of controlling an innovative damper is carried out. Due to the resistance of the granulate to the external factors, the proposed symmetrical damper can be used in difficult environmental conditions. The process of changing the underpressure and the transition state to obtain the final damping properties is very short. Therefore, the proposed structure can be used wherever there are rapid and sudden changes in the parameters of the suppressed system.

## Figures and Tables

**Table 1:** Frequency of the excitation.

*f*	$f_{1}$	$f_{2}$	$f_{3}$	$f_{4}$	$f_{5}$
Frequency [Hz]	0.4	0.9	1.5	2.1	2.8

**Table 2:** The symbols and values of the assumed underpressure.

up	up00	up01	up02	up03	up04	up05	up06	up07	up08	up09
Pressure [MPa]	0.00	0.01	0.02	0.03	0.04	0.05	0.06	0.07	0.08	0.09

**Table 3:** EPDM material properties.

Material	EPDM
Density [g/${\mathrm{cm}}^{3}$]	1.6
Hardness (Shore A)	60
Young’s modulus [MPa]	2.16
Temperature range of application [C]	−50→ 130

## Abbreviations

The following abbreviations are used in this manuscript:

SVPP	Symmetric Vacuum-Packed Particle
EPDM	ethylene–propylene–diene grains
